# Cryptic diversity in *Lithobateswarszewitschii* (Amphibia, Anura, Ranidae)

**DOI:** 10.3897/zookeys.838.29635

**Published:** 2019-04-11

**Authors:** James Cryer, Felicity Wynne, Stephen J. Price, Robert Puschendorf

**Affiliations:** 1 School of Biological and Marine Sciences, University of Plymouth, Devon, PL4 8AA, UK University of Plymouth Plymouth United Kingdom; 2 UCL Genetics Institute, Gower Street, London, WC1E 6BT, UK UCL Genetics Institute London United Kingdom; 3 Institute of Zoology, ZSL, Regents Park, London NW1 4RY, UK Institute of Zoology, ZSL London United Kingdom

**Keywords:** Área de Conservación Guanacaste (ACG), barcoding, biodiversity, CO1, phylogenetics, phylogeography, 16S

## Abstract

*Lithobateswarszewitschii* is a species of ranid frog distributed from southern Honduras to Panama. This species suffered severe population declines at higher elevations (above 500 m a.s.l.) from the 1980s to early 1990s, but there is more recent evidence of recovery in parts of its range. Here we advocate for the status of *Lithobateswarszewitschii* as a candidate cryptic species complex based on sequence data from mitochondrial genes CO1 and 16S. Using concatenated phylogenies, nucleotide diversity (K2P-π), net between group mean distance (NBGMD) (π_net_) and species delimitation methods, we further elucidate cryptic diversity within this species. All phylogenies display polyphyletic lineages within Costa Rica and Panama. At both loci, observed genetic polymorphism (K2P-π) is also high within and between geographic populations, surpassing proposed species threshold values for amphibians. Additionally, patterns of phylogeographic structure are complicated for this species, and do not appear to be explained by geographic barriers or isolation by distance. These preliminary findings suggest *L.warszewitschii* is a wide-ranging species complex. Therefore, we propose further research within its wider range, and recommend integrative taxonomic assessment is merited to assess species status.

## Introduction

Cryptic species are poorly defined and highly heterogeneous. Identification of potential singular, nominal species may be masked when morphological traits are shared within and between sister taxa (Bickford et al. 2007). Evolutionary mechanisms that produce cryptic species are also diverse and may best be explained by recent divergence, niche conservatism, and morphological convergence (Fišer et al. 2018). Although considered evidence of incomplete species inventories, or potential sources of bias within biodiversity research (Fišer et al. 2018), cryptic species are evidently common (Adams et al. 2014) and extensive among animal phyla (Perez-Ponce de León and Poulin 2016). Species concepts have been a topic of debate since Darwin’s Origin of Species (Mallet 2008), yet most contemporary biologists conceptually envisage separately evolving segments of metapopulation-level evolutionary lineages (Mayden 1997, de Queiroz 1998, 1999, Hey et al. 2003, Bock 2004, Hey 2006).

Given that the majority of species remain undescribed, endeavours to explain and catalogue biodiversity are inevitable to both understanding and preventing extinctions (Pimm et al. 2014). For amphibians especially, being the most threatened group of vertebrates (Stuart et al. 2004), identifying cryptic diversity is fundamental to their conservation. Habitat loss, fragmentation, climate change and disease epidemics have produced a global decline in amphibian populations (Baillie et al. 2004, Stuart et al. 2004). Losses reflect patterns of ecological preference, range and taxonomic association, with montane stream dwelling species most affected (Stuart et al. 2004). It is also probable that the number of amphibian species is highly underestimated (Fouquet et al. 2007a, Vieites et al. 2009).

Whereas some species are presumed to be widely distributed, those within a cryptic complex may have smaller ranges or different ecological requirements (Stuart et al. 2006), meaning failure to recognize these taxa can leave them susceptible to mismanagement. However, when genetic differentiation is established, it can unveil previously unknown units of diversity and endemism (Bickford et al. 2007) that may subsequently warrant protection or species status (Whitfield et al. 2016).

High levels of genetic diversity in Costa Rican and Panamanian frog populations are well recognized (Crawford 2003), as are cryptic species (Wang et al. 2008). *Lithobateswarszewitschii* (Ranidae) (Schmidt, 1857) is a proposed candidate species – a provisional designation pending further systematic investigation (Vieites et al. 2009). Crawford et al. (2010) (Suppl. material 1) showed that within the amphibian community at El Copé (Omar Torrijos National Park), Panama, *L.warszewitschii* displayed 14.7% pairwise divergence between conspecifics at the CO1 locus. This is an unusually high degree of polymorphism for a single species in sympatry (Crawford 2003, Vences et al. 2005), providing additional evidence this taxon likely contains candidate cryptic lineages (Mallet 2008). Paz et al. (2015) compared El Copé with allopatric populations from Brewster (Chagres National Park), revealing 11% pairwise divergence. Consequently, breeding strategy, dispersal and landscape resistance may help explain this variation between both sites.

*Lithobateswarszewitschii* occurs from Honduras to Panama and has been recorded at elevations up to 1740 meters above sea level (m a.s.l.). They are fairly common, diurnal and generally abundant frogs in forests near streams where they breed (Savage 2002). In Costa Rica, population declines occurred in montane areas such as Tapantí, Monteverde, and Braulio Carrillo (Bolaños 2002, Puschendorf et al. 2006). Post-decline it was found to be rare in San Vito (Santos-Barrera et al. 2007) and vanished but found again at San Ramón (IUCN 2015). *Lithobateswarszewitschii* was also found to be abundant at mid-elevation sites in Guayacán (Kubicki 2008), Corcovado, Ciudad Colón, and Tinamastes (IUCN 2015). A population decline also occurred at lowland site La Selva (Whitfield et al. 2007); however, it is not generally abundant at lower elevations (IUCN 2015). Pre-decline it was one of the most abundant tadpoles encountered in streams at El Copé, Panama, (Ranvestel et al. 2004), but was later extirpated following the emergence of a virulent pathogen (Crawford et al. 2010). In Nicaragua, it was found to be abundant in Río San Juan (Sunyer et al. 2009) and numbers were increasing at Quebracho (Barquero et al. 2010) post decline, although Nicaragua’s amphibian decline history is much more nebulous than Costa Rica’s. No data was found for Honduras, and additional research is needed to ascertain population sizes, distributions, trends and threats throughout its full range (IUCN 2015).

In this study we expand the research on cryptic diversity within *L.warszewitschii*, based on published sequence data from two localities in Panama (Crawford et al. 2010, Paz et al. 2015) and samples collected from the Área de Conservación Guanacaste (ACG) in northwestern Costa Rica. Using phylogenetic data, species delimitation methods and nucleotide diversity within CO1 and 16S loci we make inferences about phylogeographic structure and proposed candidate status across its wider range.

## Methods

### Field sampling

**Figure 1.**
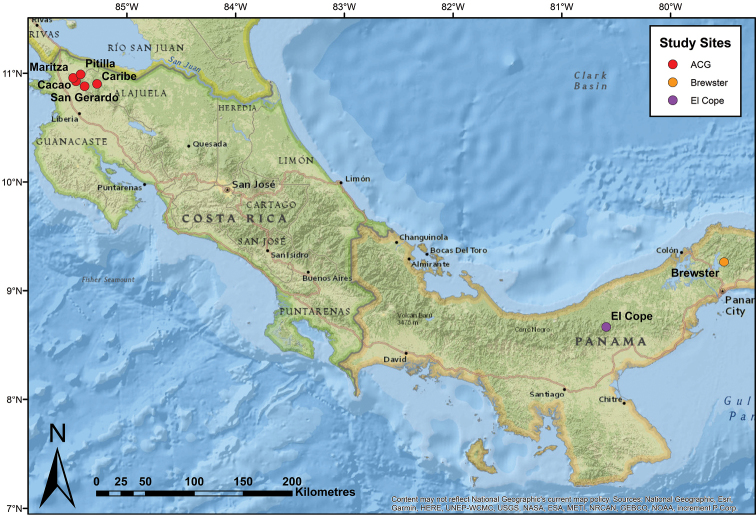
Study sites included in phylogenetic analysis of *L.warszewitschii*. Sites: Cacao, Caribe, Maritza and San Gerardo are within the Área de Conservación Guanacaste (ACG), Costa Rica. Sites El Copé and Brewster are within Panama.

*Lithobateswarszewitschii* were sampled from five field sites within the Área de Conservación Guanacaste (ACG), Costa Rica: Pitilla, San Gerardo, Maritza, Cacao, and Caribe (Figure 1; for further detail see https://www.acguanacaste.ac.cr) between June 2015 – August 2017 (Table 1). Streams and surrounding forest are preferred habitat for *L.warszewitschii* (Savage 2002), and sampling was conducted within these habitats. Each individual was captured, housed separately in moist bags (Beaupre et al. 2004), identified based on morphology (Savage et al. 2002, Leenders 2016), and toe-clipped (Perry et al. 2011). Individuals were then released back at the point of capture.

A total of 34 samples were collected from ACG and obtained from GenBank, but only 29 had both CO1 and 16S available and therefore used in this analysis. All data for *L.warszewitschii* samples collected in Panamanian sites El Copé and Brewster were obtained from other studies (Crawford et al. 2010, Paz et al. 2015).

**Table 1.** Information on study sites.

Sites	Collection dates	No. tissue samples	Habitat	Longitude /	Elevation(m)	Reference
Pitilla	August, 2016	1	Rainforest	10.989, -85.426	650–750	Field data – this study
	June, 2017	1				
San Gerardo	August, 2017	2	Rainforest/pastureland	10.881, -85.389	470–640	Field data – this study
Maritza	June, 2015	7	Dry/wetforest	10.956, -85.495	570–610	Field data – this study
	August, 2015	7				
	November, 2016	6			
	July, 2017	3				
	August, 2017	5				
Cacao	November, 2016	4	Rain/cloud forest	10.923, -85.468	980–1130	Field data – this study
	August, 2017	3				
Caribe	June, 2015	4	Rainforest	10.902, -85.275	370	Field data – this study
El Copé	July, 2010	NA	Rainforest	8.667, -80.592	700–750	(KRL0823) Paz et al. 2015
Brewster	June, 2015	NA	Rainforest	9.265, -79.508	130–810	(CH6868) Paz et al. 2015

Description of sites where populations of *Lithobateswarszewitschii* were sampled. Habitat type, georeferences, and information sources (field data GPS coordinates, or external sources, e.g., other researchers, ACG website, or literature) are included.

### Lab work

In order to extract DNA from tissue samples a standard ammonium acetate protocol was used (Nicholls et al. 2000). The Cytochrome c oxidase subunit I (CO1) and 16S ribosomal RNA (16S) mitochondrial genes were targeted for amplification by PCR. 16S primers (16Sar-L +16Sbr-H) and reaction protocols were adapted from Kessing et al. (2004). Multiple primers were used in the CO1 reactions to maximize the number of successful PCR products. CO1 primers (dgLCO-1490 + dgHCO-2198) and reaction protocols were adapted from Meyer et al. (2005) and CO1 primers (Chmf4 + Chmr4; Che et al. 2012) followed reaction protocols by Ivanova et al. (2008).

Extracted DNA from a subset of samples was sent to the Canadian Centre of DNA barcoding for PCR amplification and sequencing. These samples used CO1 primers (C_VF1LFt1 + C_VF1LRt1) in PCR reactions (Ivanova et al. 2007). The remaining samples were amplified in-house. Thermocycler (*Techne Prime Gradient*) programmes differed depending on the primer and reaction used. CO1 (dgLCO-1490 + dgHCO-2198) and 16S (16Sar-L + 16Sbr-H) reactions were run using the protocol outlined by Crawford et al. (2010). Primer set (CO1, Chmf4 + Chmr4) followed thermocycler profiles by (Ivanova et al. 2008). Two percent agar gels were used for electrophoresis with 1% TAE (Smith et al. 2008). Gels were visualized using an *ImageQuant LAS4000* and *Nanodrop 2000* quantification was performed on each successful PCR product visualized at the correct length, prior to dilution.

### Bioinformatics

Concatenated gene alignments were used in the phylogenetic analyses. GENEIOUS v11.0.5 (Kearse et al. 2012) bioinformatics software was used to assemble forward and reverse sequences from returned CO1 and 16S chromatographs. Forward and reverse (compliment) sequences were aligned using Geneious’ alignment (Global alignment with free end Gaps; Cost matrix = 65% similarity (5.0/-4.0); Gap open penalty = 12; Extension penalty = 3). Sequences were trimmed at the 3’ and 5’ ends where low quality base calls were present. Consensus sequences were produced for each sample, ranging from 609–658 base pairs (bp) in length for CO1 and 578–601bp for 16S. For both CO1 and 16S, a BLAST search (Altschul et al. 1990) was conducted using a consensus sequence derived from all Costa Rican sequences. Additional *Lithobates* species sequence data were downloaded to represent an ingroup for *L.warszewitschii* based on previous phylogenetic studies (e.g., Hillis and Wilcox 2005, Frost et al. 2006, Che et al. 2007, Huang et al. 2016): *Lithobatesclamitans* (Latreille, 1801), *Lithobatescatesbeiana* (Shaw, 1802), *Lithobatesmaculata* (Brocchi, 1877), *Lithobatespalmipes* (Spix, 1824), *Lithobatesseptentrionalis* (Baird, 1854), *Lithobatessylvaticus* (LeConte, 1825), *Lithobatesvaillanti* (Brocchi, 1877), *Ranamaoershanensis* (Lu et al., 2007) was used as an outgroup (Zhou et al. 2017). All sequences were archived in Genbank (Benson et al. 2012; Table 2). All relevant sequences for each gene were then Geneious aligned (Maddison 1997). Only individuals which had sequence data for both genes were included in the concatenated alignment for the phylogenetic analyses. *Lithobatesclamitans*, *L.maculata*, *L.septentrionalis* and *L.vaillanti* were represented by different individuals on 16S and CO1 phylogenetic analyses.

**Table 2.** Genbank (NCBI) Voucher ID & Accession numbers.

Species	Study site	Voucher ID	CO1 Genbank Accession #	16S Genbank Accession #
* L. warszewitschii *	Maritza	RP 388	MH559513	MH603380
	Maritza	RP 389	MH559517	MH603379
	Pitilla	RP 435	NA	MH603378
	San Gerardo	RP 466	MH559519	MH603377
	San Gerardo	RP 475	MH559514	MH603376
	Maritza	RP 496	MH559518	MH603375
	Maritza	RP 500	MH559515	MH724925
	Cacao	RP 878	NA	MH724926
	Cacao	RP 885	MH559516	MH724927
	Cacao	RP 887	NA	MH724928
	Caribe	RP Fw142	MH559500	MH603393
	Caribe	RP Fw144	MH559501	MH603392
	Caribe	RP Fw147	MH559502	NA
	Maritza	RP Fw455	MH559503	MH603391
	Maritza	RP Fw457	MH559504	MH603390
	Pitilla	RP Fw570	MH559505	MH603389
	Cacao	RP Fw591	MH559506	MH603388
	Cacao	RP Fw597	MH559507	MH603387
	Cacao	RP Fw601	MH559508	MH603386
	Cacao	RP Fw616	NA	MH603385
	Maritza	RP Fw618	MH559509	MH603384
	Maritza	RP Fw619	MH559510	MH603383
	Maritza	RP Fw620	MH559511	MH603382
	Maritza	RP Fw635	MH559512	MH603381
	Brewster	CH6868	KR863019	KR863275
	Brewster	AJC1794	KR863021	KR863277
	Brewster	AJC1798	KR863026	KR863282
	Brewster	CH6658	KR863027	KR863283
	Brewster	CH6659	KR863028	KR863284
	El Copé	KRL 0823	FJ766749	FJ84384
	El Copé	KRL 1540	FJ766751	FJ84552
	El Copé	KRL 1508	KR911913	KR911916
	El Copé	KRL 1496	KR911914	KR911917
	El Copé	KRL 1567	KR911915	KR911918
* L. catesbeiana *	*NA*	–	KX686108*	KX686108*
* L. clamitans *	*NA*	–	EF525879	KY677813
* L. maculata *	*NA*	–	*NA*	AY779207
* L. palmipes *	*NA*	CFBHT12435	KU494586	KU495379
* L. septentrionalis *	*NA*	–	EF525896	AY779200
* L. sylvaticus *	*NA*	–	KP222281*	KP222281*
* L. vaillanti *	*NA*	–	KY587190	AY779214
* R. maoershanensis *	*NA*	SYNU08030061	KX1397728	KX1397722

Voucher ID and GenBank accession numbers for all individuals and sequences of *Lithobateswarszewitschii* used in this study. (*) indicates that gene sequences derived from a whole mitochondrial genome sequence.

Separate Bayesian consensus trees for the CO1 and 16S alignments were estimated independently using MR BAYES v3.2.6 (Ronquist et al. 2013) to ensure they did not conflict with each other. After establishing that there were no conflicts, columns with gaps were removed from the two individual alignments, which were then concatenated end to end with PhyUtility v.2.7.1 (Smith et al. 2008). This concatenated alignment was then used to construct trees using a Bayesian framework (Mr. Bayes with default settings used for Markov chain Monte Carlo (MCMC) analysis—1,000,000 generations, 4 chains, 2 runs, a sample frequency of 500, and a 25% burn-in) and a maximum likelihood framework (RAxML; Stamatakis 2014); 20 maximum-likelihood trees generated on distinct starting trees, 1000 bootstrap replicates calculated and annotated on the best maximum-likelihood tree). The alignment was partitioned by gene, meaning model parameters were unlinked across the partition, to account for the different evolutionary histories of the CO1 and 16S genes. The General Time Reversible (GTR) model of substitution (Tavaré 1986) was used for all trees in order to be consistent between the Bayesian and maximum likelihood approaches since GTR is the model implemented in RAxML. Rate variation among sites was modelled as a discrete gamma distribution with four rate categories. Trees were rooted on the outgroup (*R.maoershanensis)* and visualised in FigTree v1. 4. 2 (Rambaut 2014).

Species boundaries were assessed in two ways. The first using the GENEIOUS plugin SPECIES DELIMITATION (Masters et al. 2011), which calculates the probability of reciprocal monophylly against the null model of random coalescence (Rosenberg 2007) for single panmictic populations (Rodrigo et al. 2008) and presents the probability for correct identification for putative species, given the data (Ross et al. 2008). Groups with P (Randomly Distinct) values of 0.05 – 1, represent branching events that would be expected under a coalescent model in a Wright-Fisher population and a strict molecular clock (Rodrigo et al. 2008, Masters et al. 2011). The second method used the Automatic Barcode Gap Discovery for primary species delimitation (ABGD; Puillandre et al. 2012) via a web interface (http://wwwabi.snv.jussieu.fr/public/abgd/). A maximum of ten, and minimum of two samples per geographic locality of the focal species were used as required for the minimum estimation of genetic divergence (Hickerson et al. 2007), a minimum of one sample was considered adequate for interspecific analysis (Aliabadian et al. 2009). Where possible, the same individuals were used in the analyses of both genes. Intraspecific and interspecific genetic distances were also calculated and analysed. Average, K2P-corrected (Kimura 1980) pairwise distance (K2P-π) and net between group mean distance (NBGMD) (π_net_) (Nei and Li 1979) were calculated in MEGA v6 (Tamura et al. 2013) to assess nucleotide diversity (**π**) and cryptic speciation within and between sites.

## Results

### Phylogenetic comparison

**Figure 2.**
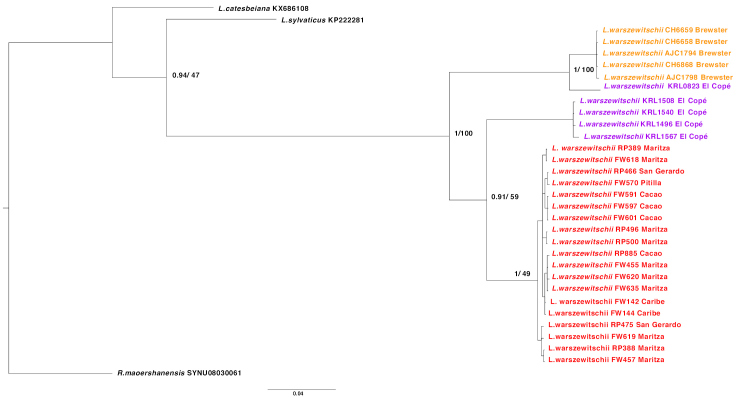
Phylogenetic reconstruction of *Lithobateswarszewitschii* relationships between Costa Rican and Panamanian populations using concatenated alignments of CO1 and 16S. Node support values (posterior probabilities) and percentages calculated from 1000 bootstrap replicates are annotated at nodes. Samples collected in different localities are represented by different colours: individuals from Área de Conservación Guanacaste (ACG; Cacao, Caribe, Maritza, Pitilla, and San Gerardo) highlighted in red, individuals from Brewster highlighted in purple, and individuals from El Copé highlighted in orange. Sample information can be found in Table 2. Separate trees were constructed in Mr. Bayes and RAxML using a GTR model of molecular evolution, both with similar topologies, therefore node supports were included in a single tree. Scale of branch lengths is in nucleotide substitutions per site.

Concatenated phylogenetic trees reconstructed using Bayesian inference and Maximum likelihood (Figure 2) methods, show similar topology of three major clades within the focal species. Geographic samples from ACG and Brewster formed well-supported independent monophyletic groups. However, samples from El Copé presented a polyphyletic structure. Four out of five individuals (KRL 1496, KRL 1508, KRL 1540, KRL 1567) formed an independent clade, sister to the ACG clade, whereas sample KRL 0823 formed a clade with samples from Brewster – revealing the presence of two taxa at El Copé. Subsequently, three clades are recognized: ACG and El Copé, containing samples exclusively from these areas, and Brewster (including sample KRL 0823 from El Copé). Single gene trees showed a similar topology to the concatenated ones (Suppl. material 1: Figures S1, S2).

### CO1 operational taxonomic units (OTUs) delimitation results

CO1 species delimitation in GENEIOUS yielded three OTUs (Table 3). Focal clades ACG, Brewster (+KRL 0823), and El Copé (KRL 1496, KRL 1508, KRL 1540, KRL 1567) had P values <0.05, indicating they are not conforming to the expected Wright-Fisher criteria. According to this assumption and the data present, all clades were taxonomically distinct. ABGD analysis identified four OTUs within *L.warszewitschii*, with KRL 0823 forming its own OTU (p= 0.0359). ABGD also supported the three distinct OTUs outlined by species delimitation in GENEIOUS (p= 0.0599, Suppl. material 1: Table S1 and Suppl. material 1: Figure S3).

**Table 3.** CO1 Species delimitation results.

OTU	Closest OTU	Monophyletic?	Intra Dist	Inter Dist – Closest	Intra/Inter	PID(Strict)	PID(Liberal)	Av(MRCA-tips)	P(Randomly Distinct)	Rosenberg’sP(AB)
1: ACG	2: El Copé	yes	0.01	0.109	0.08	0.97 (0.91,1.0)	0.99 (0.96,1.0)	0.0076	0.05	8.10E-06
2: El Copé	1: ACG	yes	0.01	0.109	0.06	0.83 (0.69,0.97)	0.97 (0.86,1.0)	0.0047	0.05	8.10E-06
3: Brewster & KRL 0823	2: El Copé	yes	0.02	0.197	0.08	0.88 (0.75,1.0)	0.97 (0.87,1.0)	0.0211	0.05	1.10E-07
4: *L.palmipes*	5: *L.vaillanti*	yes	0	0.114	0	0	0.96 (0.83,1.0)	0	NA	1
5: *L.vaillanti*	4: *L.palmipes*	yes	0	0.114	0	0	0.96 (0.83,1.0)	0	NA	1
6: *L.catesbeiana*	7: *L.clamitans*	yes	0	0.057	0	0	0.96 (0.83,1.0)	0	NA	1
7: *L.clamitans*	* L. catesbeiana *	yes	0	0.057	0	0	0.96 (0.83,1.0)	0	NA	1
8: *L.septentrionalis*	7: *L.clamitans*	yes	0	0.092	0	0	0.96 (0.83,1.0)	0	NA	0.33
9: *L.sylvaticus*	8: *L.septentrionalis*	yes	0	0.238	0	0	0.96 (0.83,1.0)	0	NA	0.17

Species delimitation results of *Lithobateswarszewitschii* in Costa Rica and Panama using partial sequences of the CO1 gene. Analysis conducted in Geneious using the Species Delimitation plugin (Masters et al. 2011). Clades defined in phylogenetic analysis: ACG, Brewster (+ sample KRL 0823) and El Copé are all represented as putative species. The table also includes ingroup and outgroup species.

### CO1 and 16S nucleotide diversity

K2P-π at the CO1 and 16S loci showed a mean value of 7.2% and 3.4%, respectively, within all *L.warszewitschii* samples (Table 4). Samples from El Copé had the highest intra-group mean distance at 6.3% and 3.2%, respectively, whereas samples from ACG had 0.4% and 0.3% and within Brewster 0.1% and 0.2%, respectively. Mean intraspecific distances between ACG and Brewster samples (CO1/16S) were the highest at 15.7%/7.2% (Suppl. material 1: Tables S2, S3). Samples from ACG and El Copé shared the lowest distance at 10.7%/6.2%, and the intermediate distance was 13.8%/6.7% between Brewster and El Copé samples. Interspecific comparisons within the genus resulted in lower interspecific distances among recognized species (COI/16S), such as: *L.clamitans* and *L.catesbeiana* (5.7%/2%), *L.septentrionalis* and *L.clamitans* (8.3%/3.1%), *L.septentrionalis* and *L.catesbeiana* (8.6%/2.2%).

**Table 4.** Intraspecific nucleotide diversity (**π**) within geographic groups of *L.warszewitschii*.

Population	Mean(π)	Range(π)
	CO1	
ACG	0.004	0-0.008
El Copé	0.063	0.002-0.154
Brewster	0.001	0-0.002
* L. warszewitschii *	0.072	0-0.166
	**16S**	
ACG	0.003	0-0.009
El Copé	0.032	0-0.076
Brewster	0.002	0-0.006
* L. warszewitschii *	0.034	0-0.079

Nucleotide diversity (**π**) within *Lithobateswarszewitschii* for the geographic groups ACG, Brewster and El Copé based on pairwise values for CO1 and 16S sequences. Analyses were conducted using the Kimura 2-parameter model (Kimura 1980). The rate variation among sites was modelled with a gamma distribution (shape parameter = 4).

### CO1 and 16S Net between group mean distance (NBGMD) (π_net_)

At the CO1 and 16S loci the largest NBGMD (π_net_) was 15.4% and 6.9%, respectively, between ACG and Brewster samples (Suppl. material 1: Tables S2, S3). Samples from ACG and El Copé shared the lowest distance at 7.3% and 4.5%, respectively, and the intermediate distance was 10.6% and 5%, respectively, between El Copé and Brewster samples. Most intraspecific distances between the geographic groups within *L.warszewitschii*, surpassed the interspecific values between recognized species within the genus (CO1/16S), such as: *L.catesbeiana* and *L.clamitans* (5.7%/2%), *L.clamitans* and *L.septentrionalis* (8.3%/3.1%), *L.catesbeiana* and *L.septentrionalis* (8.6%/2.2%).

### Discussion

The concatenated phylogenetic trees consistently outlined three distinct clades within *Lithobateswarszewitschii* supported by high posterior probabilities, bootstrap values, and taxonomic distinctness at the CO1 locus. No field sites within the ACG exhibited any well-defined cladistic structure, indicating it is a larger panmictic population. The individuals from El Copé were polyphyletic, revealing the presence of two OTUs at this site. Geographic groups within *L.warszewitschii* also exhibited greater genetic distances than many other recognized species pairs within the genus, suggesting cryptic species may be present.

In the analyses of nucleotide diversity and NBGMD, isolation by distance (IBD) (Wright 1943) does not explain all patterns of genetic variation, as samples from ACG and El Copé are most closely related in all scenarios. Additionally, the range of 16S (K2P-π) distance values within El Copé reached the highest for any geographic group at both loci. Thus, there is evidence that IBD contributes towards greater polymorphism in the most isolated allopatric populations, but other intrinsic (dispersal capability) and extrinsic (environmental and ecological) factors may explain large variation within and between finer geographic scales.

Isolation by distance may be the main driver of divergence or speciation among conspecific populations (Slatkin 1993) in allopatry (Vences and Wake 2007), other drivers include, low vagility due to limitations of physiology (Balinsky 1981, Navas and Otani 2007) and dispersal (Blaustein et al. 1994). However, recurrent hybridization, secondary contact, or overlap with sister species can decrease this genetic distance correlation (Fouquet et al. 2007b). If populations follow a simple pattern of IBD, they may be considered with some probability, conspecific (Fouquet et al. 2007a). Conversely, where large variations in genetic distance cannot be explained by this concept, it is likely that cryptic speciation is present.

*Lithobateswarszewitschii* is widely distributed throughout Central America, and the possibility of vicariance may explain mechanisms for genetic divergence. The Talamanca mountain range divides the Pacific and Atlantic versants at ~2000m altitude (Savage 1982). Many of the Isthmian fauna disperse through the Caribbean lowlands but have disjunct distribution along Costa Rica’s Pacific southwest (McDiarmid and Savage 2005) that historically contained more dry forest. Crawford et al. (2007) hypothesized that the presence of a filter barrier (Remington 1968), caused by extreme topography and narrowing of the rainforest corridor in Panama’s Bocas del Toro province induced the deepest phylogeographical split between northern and southern populations of *Craugastor* rainforest species. For *Craugastorfitzingeri* (Schmidt, 1857), a generalist species, these effects were much less accentuated and its phylogenetic structure may be attributed to a more recent range expansion. For *L.warszewitschii*, gene flow is still possible, even if regional dry forests were transformed into savannah during the Pleistocene glacial maxima (Piperno and Pearsall 1998), patches of gallery forest that allowed reproduction in freshwater could permit dispersal westward into Costa Rica.

Although vicariance does divide sister species (Avise et al. 1987), it fails to form a general explanation for divergence in the tropics (Antonelli et al. 2010). Barriers such as mountains do not impede gene flow directly, but promote ecological gradients (Janzen 1967). An alternative explanation for the phylogeographic structure within *L.warszewitschii* could be peripatric (Mayr 1954) or dichopatric (Bush 1994) speciation – a common mode of evolution in amphibians (Vences and Wake 2007).

Paz et al. (2015) used a trait-based phylogeographic approach to model environmental and ecological variables in Panamanian frog populations. Indirect development encouraged greater dispersal and species with large ranges had lower genetic divergence – a characteristic associated with generalists (Duminil et al. 2007). Despite being oviparous and wide-ranging, *L.warszewitschii* scored highest when modelling landscape resistance (resistance to dispersal caused by environmental conditions) and was highly divergent between Brewster and El Copé, with large genetic distances in proportion to their geographical distance. A possible explanation for this pattern could be a secondary contact during the post-glacial maxima (Schneider 1993) or selection for different ecological roles, such as within habitat or resource use (Alizon et al. 2008). It is true that *L.warszewitschii*’s colouration, habitat use, elevation range, and distribution vary (Savage 2002, Leenders 2016). Thus, high intraspecific diversity may be attributed to ecological specialization (Schluter 2000) in allopatry or coexistence of sister species in sympatry, such as in El Copé. For example, even if broad colouration of this species is genuine, frogs use non-morphological signals such as advertisement calls, cuticular hydrocarbons and other pheromones in mating systems and species recognition (Bickford et al. 2007), meaning they often remain inconspicuous. Divergent or cryptic species should therefore be considered a hypothesis of separately evolving entities (Hey et al. 2003, de Quieroz 2007, Fiser et al. 2018) and species status further scrutinized through integrative taxonomic methods (Padial et al. 2010).

Polyphyly can be used as indication of undescribed species in a lineage (Fouquet et al. 2007a). However, its presence complicates the classification of species in phylogenies as it may represent transitional stages in the evolution of taxa (Hörandl and Stuessy 2010, Xiang et al. 2012). Cryptic species often show morphological, ecological or genetic differentiation and usually a degree of reproductive isolation, which may occur through phenotypic plasticity or single locus polymorphisms. Hybridization may persist, leaving traces of introgression, speciation or hybrid vigour. Alternatively, fusion may be resisted by disruptive/divergent selection or postzygotic isolation (Sasa et al. 1998). This continuum is evident across large geographic ranges to highly localized areas, providing explanations for the evolutionary transitions of ecological races to species (Mallet 2008). Consequently, in *L.warszewitschii*, patterns of polyphyly, relatedness between ACG and El Copé samples, or large pairwise ranges in sympatry may reflect occasional or historical gene flow from migrants, hybridization, introgression, retention of ancestral polymorphisms or incomplete lineage sorting when using mitochondrial genes (Moritz and Cicero 2004). Alternatively, the presence of two sympatric OTUs at El Copé, may reflect human-induced introduction. Because of these scenarios, nuclear DNA is also recommended in subsequent evolutionary and taxonomic studies (Vences et al. 2005).

At both CO1 and 16S loci, K2P-π mean (Meyer and Paulay 2005) intraspecific ingroup values overlapped with interspecific species values, surpassing proposed general thresholds: 8% at CO1 and 2% 16S (Crawford et al. 2010), 10% CO1, 5% 16S (Vences et al. 2005) and for neotropical amphibians at 16S (>3%) (Fouquet et al. 2007a). This indicates a wider ranging cryptic complex is present, and advocates for the use of both genes in comparative amphibian phylogenetics (Vences et al. 2005). Ultimately, concatenated genes may yield the best phylogenies (Gadagkar et al. 2005), however, interspecific comparisons are limited in this study due to having one individual representing each congeneric species, and an incomplete taxonomy that can hamper results (Meyer and Paulay 2005).

## Conclusions

The type specimen of *Lithobateswarszewitschii* originated from Volcán Chiriqui, western Panama (Schmidt 1857, Savage 1970), a locality near the Costa Rican border at almost equal distance between ACG and Brewster. Whilst the topotype locality was not sampled, all clades in this study may represent cryptic species. We have extended the research on cryptic diversity within *L.warszewitschii* by revealing an additional clade from ACG, and propose this clade is a candidate cryptic species that warrants further taxonomic investigation. Determination of evolutionary mechanisms are beyond the scope of this study, but an additional paraphyletic lineage from Costa Rica suggests it is probably a wide-ranging species complex, a likely scenario for many neotropical amphibians. Population trends in Costa Rica and Panama reflect both historical factors and recent habitat destruction, declines and introduced disease. Further sampling within Costa Rica, Nicaragua, and Honduras is likely to yield more cryptic diversity, and extirpation of a candidate lineage within El Copé (Crawford et al. 2010) highlights the importance of DNA barcoding in rapid, preliminary species identification. Such assessments are necessary to inform biodiversity estimates, taxonomic progress, and conservation of amphibian species. Phylogeographic structure in *L.warszewitschii* highlights the difficulty in explaining mechanisms of speciation in Mesoamerican amphibian fauna. Evolutionary theory, supported by morphological, ecological, physiological and multiple genetic methods are necessary to evaluate divergent processes in this group, and in achieving species status of sister taxa in this complex.

## Supplementary materials

Supplementary material 1 Supplementary tables and figures Data type: molecular data Explanation note: Table S1. ABGD analysis from CO1 using all species presented in Table S2. Table S2. Estimates of evolutionary divergence (π), and net evolutionary divergence (πnet) over CO1 sequence pairs between groups. Table S3. Estimates of evolutionary divergence (π), and net evolutionary divergence (πnet) over 16S sequence pairs between groups. Figure S1. CO1 phylogenetic tree. Geographic populations ACG (red), Brewster (orange), El Copé (purple) of *L.warszewitschii* are represented. Figure S2. 16S phylogenetic tree. Geographic populations ACG (red), Brewster (orange), El Copé (purple) of *L.warszewitschii* are represented. Figure S3. Prior intraspecific genetic divergence and number of OTUs using the ABGD algorithm. This dataset is made available under the Open Database License (http://opendatacommons.org/licenses/odbl/1.0/). The Open Database License (ODbL) is a license agreement intended to allow users to freely share, modify, and use this Dataset while maintaining this same freedom for others, provided that the original source and author(s) are credited.James Cryer, Felicity Wynne, Stephen J. Price, Robert Puschendorf
