# Supplementary material for: Cryptic diversity in Lithobateswarszewitschii (Amphibia, Anura, Ranidae)
Source: Zookeys. 2019 Apr 11;838:49–69. doi: 10.3897/zookeys.838.29635 (PMC6477815; doi:10.3897/zookeys.838.29635)
Supplement: Supplementary material 1 [file zookeys-838-049-s001.doc]

Supplementary Section

Supplementary Table 1. ABGD analysis from CO1 using all species presented in Table 2.

Supplementary Table 1. The parameters include Gap width (X) = 1, (min) DIST = 0.001 - 0.1 (Max) DIST for P (P = maximum value for intraspecific divergence). Generated through the ABGD user interface website (http://wwwabi.snv.jussieu.fr/public/abgd/).

Supplementary Table 2. Estimates of evolutionary divergence (**π**), and net evolutionary divergence (π_net_) over CO1 sequence pairs between groups

Supplementary Table 2. The number of base substitutions per site from averaging over all CO1 sequence pairs between groups are shown above (**π**). The number of base substitutions per site from estimation of net average between groups of CO1 sequences are shown below (NBGMD / π_net_). Standard error estimate(s) are shown above the diagonal. Analyses were conducted using the Kimura 2-parameter model (Kimura 1980). The rate variation among sites was modelled with a gamma distribution (shape parameter = 4). The analysis involved 37 nucleotide sequences. There were a total of 658 positions in the final dataset.

Supplementary Table 3. Estimates of evolutionary divergence (**π**), and net evolutionary divergence (π_net_) over 16S sequence pairs between groups

Supplementary Table 3. The number of base substitutions per site from averaging over all 16S sequence pairs between groups are shown above (**π**). The number of base substitutions per site from estimation of net average between groups of 16S sequences are shown below (NBGMD / π_net_). Standard error estimate(s) are shown above the diagonal. Analyses were conducted using the Kimura 2-parameter model (Kimura 1980). The rate variation among sites was modelled with a gamma distribution (shape parameter = 4). The analysis involved 42 nucleotide sequences. There were a total of 601 positions in the final dataset.

1. CO1 Phylogenetic tree

Supplementary figure 1. CO1 phylogenetic tree. Geographic populations ACG (red), Brewster (orange), El Cope (purple) of *L. warszewitschii* are represented. Samples include GenBank voucher ID, NCBI database information for other ingroup/outgroup species can be found in Table. 4. Posterior probability/branch support is also shown.

1. 16S Phylogenetic tree

Supplementary figure 2. 16S phylogenetic tree. Geographic populations ACG (red), Brewster (orange), El Cope (purple) of *L. warszewitschii* are represented. Samples include GenBank voucher ID, NCBI database information for other ingroup/outgroup species can be found in Table. 4. Posterior probability/branch support is also shown.

Supplementary figure 3. Prior intraspecific genetic divergence and number of OTUs using the ABGD algorithm

Supplementary figure 3. A plot depicting how the number of OTUs recovered by the ABGD algorithm varies with increasing prior intraspecific divergence, using CO1 sequence data from this study (Table 4).
